# From demyelination to neurodegeneration in multiple sclerosis: reassessing the role of visual evoked potential P100-N145 amplitudes: a missing piece of the puzzle?

**DOI:** 10.3389/fneur.2025.1649998

**Published:** 2025-09-29

**Authors:** Nurhan Kaya Tutar, Nilufer Kale

**Affiliations:** Department of Neurology, Bagcilar Training and Research Hospital, Istanbul, Türkiye

**Keywords:** multiple sclerosis, VEP, neurodegeneration, P100–N145, disability progression, EDSS

## Abstract

**Background:**

Multiple sclerosis (MS) is a chronic demyelinating disease with a heterogeneous clinical course, making long-term disability prediction challenging. Visual evoked potentials (VEPs), particularly amplitude-based parameters, may serve as sensitive biomarkers of neurodegeneration and functional decline undetected by conventional clinical measures.

**Objective:**

To assess the relationship between longitudinal changes in P100–N145 amplitude and concurrent Expanded Disability Status Scale (EDSS) changes in relapsing–remitting MS (RRMS) and the relative utility of baseline and longitudinal VEP parameters in representing disability status.

**Methods:**

In this retrospective cohort study, 45 MS patients (90 eyes) with available VEP and EDSS data were followed for a median period of 48 months. The primary endpoints were (1) change in EDSS score over time and (2) EDSS progression, defined as any increase in EDSS score from baseline to follow-up. Generalized estimating equations (GEE) and logistic regression were used to investigate the relationships between the VEP parameters at baseline and follow-up and EDSS progression, accounting for inter-eye correlation; partial correlation analysis assessed amplitude–EDSS change associations, controlling for age.

**Results:**

EDSS progression was observed in 17.8% of patients. A longitudinal decrease in P100–N145 amplitude from baseline to follow-up was significantly associated with EDSS progression (OR: 1.511, 95% CI, *p* < 0.001). In addition, partial correlation analysis adjusting for age revealed a significant negative association between the difference in P100–N145 amplitude and EDSS difference (defined as baseline minus follow-up) in both eyes (right eye: *r* = −0.339, *p* = 0.024; left eye: *r* = −0.406, *p* = 0.006). In contrast, the changes in P100 latency and N75–P100 amplitude did not correlate significantly with EDSS worsening. Baseline VEP parameters, including P100 latency, N75–P100, and P100–N145 amplitudes, did not predict EDSS progression or change over time (all *p* > 0.05).

**Conclusion:**

Our results demonstrate that a reduction in P100–N145 amplitude over time is associated with worsening disability in RRMS. This suggests that the P100–N145 may be an underestimated marker of progressive functional deterioration in RRMS.

## 1 Introduction

Multiple sclerosis (MS) is an autoimmune disease that involves both neuroinflammation and neurodegeneration (1). Clinically, the conventional dichotomy of relapse and progression only partially reflects the underlying biological complexity of the disease. While inflammation is usually associated with relapses and neurodegeneration with disease progression, this categorization is neither exclusive nor complete. Neurodegenerative changes can also be observed during periods of clinical relapses, and conversely, inflammatory activity can persist during progressive phases despite the absence of relapses (2). The current approach provides us with the insight that the fundamental cause of irreversible disability is not only the lack of adequate recovery from relapses, but also the subclinical activity that can occur even before the first clinical findings appear.

The visual pathway is a topographically well-defined region that is susceptible to demyelination and can be affected both clinically and subclinically in MS (3). Recent revisions to the MS diagnostic criteria have emphasized the inclusion of the optic nerve as a relevant spatial extension and highlighted its importance in the early diagnosis of the disease (4). Advances in structural and functional imaging techniques have significantly improved our ability to assess the integrity of the visual pathway and have provided important insights into the pathophysiological mechanisms underlying neurological disability in MS, particularly through early detection of subclinical damage and monitoring of disease progression over time (5).

Visual evoked potentials (VEPs) are non-invasive electrophysiological techniques used to assess the functional integrity of the visual pathway, particularly the optic nerve (6). They are typically elicited with pattern reversal stimuli and produce a triphasic waveform comprising the N75, P100, and N145 components—a negative peak at approximately 75 ms, a positive peak around 100 ms, and a second negative peak at 145 ms. In clinical practice, latency and amplitude are the two primary VEP parameters, each providing information about different aspects of MS pathology. While latency prolongation is typically associated with demyelination and slowed conduction, a decrease in amplitude more often reflects axonal loss or more extensive neurodegeneration (7). In addition to conventional measurements of overall latency and amplitude, examination of specific segments of the VEP waveform can provide information on various aspects of visual pathway involvement in MS. The N75–P100 interval reflects early visual cortical activation and appears to be more sensitive to demyelination-induced conduction delay. In contrast, the P100–N145 segment can capture ongoing cortical processing and provide information about axonal integrity (8). Analyzing these segments separately can help to reveal more subtle aspects of disease progression that often go unnoticed when only overall latency or amplitude values are used.

In this study, we took a segment-based approach and analyzed P100–N145 amplitude, N75–P100 amplitude, and P100 latency to understand what each segment might reveal about the underlying disease process. Our aim was not to emphasize one parameter over the others, but to explore how these parameters might complement each other by reflecting different stages or aspects of neurodegeneration. By correlating these VEP measures with the Expanded Disability Status Scale (EDSS), we aimed to determine how various aspects of visual pathway dysfunction relate to long-term neurodegenerative changes in MS.

## 2 Methods

### 2.1 Participants

This retrospective cohort study included patients diagnosed with relapsing–remitting MS (RRMS) between 2017 and 2024, based on the 2017 revised McDonald criteria (9). Inclusion criteria were: (1) age >18 years, (2) fulfillment of the 2017 McDonald diagnostic criteria for MS, (3) availability of both baseline and follow-up VEP recordings, and (4) no clinical relapses or treatment changes between the two VEP assessments. The first VEP could have been performed shortly after a clinical relapse, but only patients with a stable clinical course and unchanged treatment during the VEP-to-VEP interval were included. Patients with comorbidities affecting the visual system (e.g., glaucoma, diabetic retinopathy) were excluded to minimize confounding effects on VEP amplitudes. Eyes with a documented history of clinically diagnosed optic neuritis (whether treated or untreated) were also excluded from the analysis. Both eyes were included in the analysis when they met the eligibility criteria. The study was approved by the local ethics committee and conducted in accordance with the Declaration of Helsinki. Written informed consent was obtained from all participants.

### 2.2 Data collection

**Figure 1:**
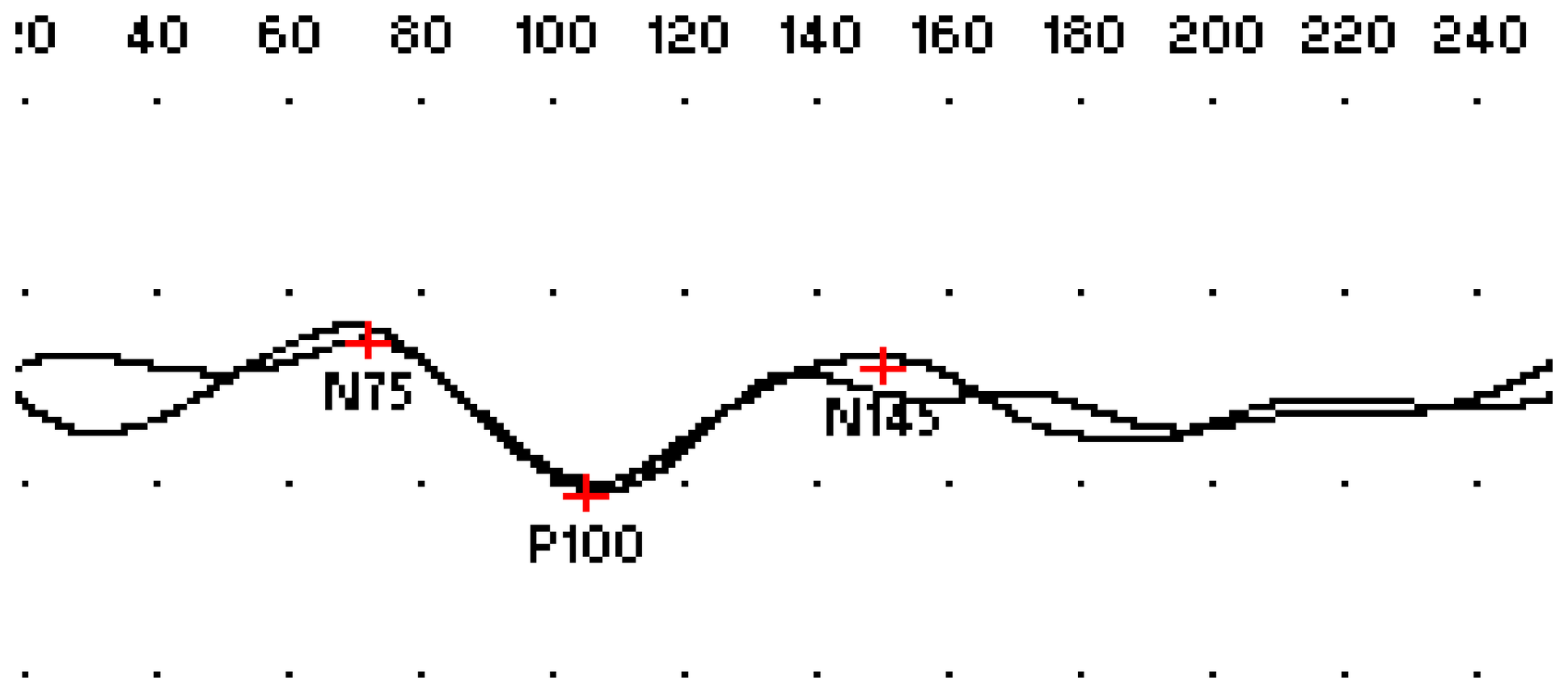
Representative waveform of the visual evoked potential (VEP) with reversal of the pattern showing the principal components: N75 (first negative deflection), P100 (major positive peak around 100 ms) and N145 (second negative deflection). The peaks are marked to identify the latency, and the amplitude measurements are performed between these components (N75–P100 and P100–N145).

Demographic and clinical data, including age, sex, disease duration, EDSS scores, and disease-modifying treatment (DMT) status (no treatment, first-line, or second-line therapy), were retrospectively extracted from patient records. To ensure temporal consistency between the clinical and electrophysiological assessments, the EDSS scores corresponding to the clinical visits during which the VEP recordings were obtained were used for the analysis (10). The VEP parameters analyzed included P100 latency, N75–P100 amplitude, and P100–N145 amplitude (Figure 1). VEP recordings were performed using a standard pattern-reversal protocol, in accordance with the guidelines of the International Society for Clinical Electrophysiology of Vision (ISCEV) (11). The primary endpoint was EDSS progression, measured as the difference in EDSS scores between baseline minus final follow-up. Secondary analyses examined the relationship between changes in VEP parameters and binary EDSS worsening (defined as any change in EDSS from baseline).

### 2.3 Statistical analysis

The data were analyzed using SPSS version 23.0. Generalized estimating equations (GEE) were used to compare the quantitative variables between the treatment groups, taking into account the correlation between the individual subjects. Partial correlation analysis was used to assess the relationships between continuous variables, with age as a covariate. As each patient contributed data from both eyes, the right and left eyes were analyzed separately in order to avoid dependencies between the subjects. The influence of independent variables on EDSS change was analyzed using regression analysis within GEE. In addition, patient-specific characteristics were entered into the GEE models as covariates to control for inter-individual variability. GEE was also applied with a binary logistic link function to examine the predictors of EDSS deterioration. Quantitative data were expressed as mean ± standard deviation for normally distributed variables and as median (minimum-maximum) for non-normally distributed variables. A significance level of *p* < 0.05 was considered statistically significant.

## 3 Results

A total of 45 patients with RRMS, contributing 90 eyes, were included in the study. The median age of the cohort was 35 years, and 75.6% were female. The median follow-up duration was 48 months (range: 15–85 months), and the median interval between the two VEP assessments was 28 months (range: 5–67 months). In terms of treatment status, 4.4% of patients were untreated, 55.6% were receiving first-line therapies, 24.4% were naive to second-line therapies, and 15.6% had switched to a second-line therapeutic agent. During the follow-up period, EDSS progression was observed in 17.8% of patients, while 82.2% remained clinically stable. At baseline, the median EDSS score was 1.5 (range: 1–6), with a slight increase to 1.5 (range: 1–6.5) at follow-up (Table 1).

**Table 1:** Distribution of demographic and clinical findings of patients.

	Frequency	Percentage
Gender		
Female (%)	34	75.6
Male (%)	11	24.4
Treatment		
None	2	4.4
First-line therapy (%)	10	55.6
Naive second-line therapy (%)	11	24.4
Switch to second-line therapy (%)	7	15,6
EDSS worsening		
No	37	82.2
Yes	8	17.8
	Median (min-max)	
Age (years)	35 (20–58)	
Follow-up duration (months)	48 (15–85)	
Baseline EDSS	1.5 (1–6)	
Follow-up EDSS	1.5 (1–6.5)	
Interval between VEPs (months)	28 (5–67)	
Baseline P100 latency (ms)	107 (94–184)	
Baseline N75–P100 amplitude (μV)	7.92 (3.19–16.6)	
Baseline P100–N145 amplitude (μV)	9.155 (1.2–17.9)	
Follow-up P100 latency (ms)	114 (91–160)	
Follow-up N75–P100 amplitude (μV)	7.975 (2.43–24.5)	
Follow-up P100–N145 amplitude (μV)	8.425 (1.23–27.1)	

VEP, visual evoked potentials; EDSS, Expanded Disability Status Scale. n (%), median (minimum–maximum).

### 3.1 VEP findings

At baseline, the median P100 latency was 107 ms, the median N75–P100 amplitude was 7.92 μV, and the median P100–N145 amplitude was 9,155 μV. At follow-up, the median P100 latency increased to 114 ms, the median N75–P100 amplitude increased to 7,975 μV, and the median P100–N145 amplitude decreased to 8,425 μV (Table 1).

### 3.2 Associations between VEP parameters and disability status

According to the GEE analysis, at baseline, patients in the untreated group had significantly lower EDSS scores than those who had been switched to second-line therapy (mean difference: −1.85 points, *p* < 0.001). EDSS scores were also significantly lower in the first-line treatment group (−1.445 points, *p* < 0.001) and in second-line naive patients (−1.387 points, *p* < 0.001) compared to the switch group. Each 10 ms increase in baseline P100 latency was associated with a 0.19-point increase in baseline EDSS (*p* = 0.001), while no other covariates were significantly related to baseline EDSS (*p* > 0.050) (Table 2).

**Table 2:** Regression analysis results for the EDSS variable at the time of the first VEP assessment.

	EDSS at the time of initial VEP			
	β1 (%95 CI)	SD	Test statistic	*p*
Constant	0.866 (−0.921 to 2.653)	0.912	0.903	0.342
Eye				
Right	0.01 (−0.217 to 0.236)	0.116	0.007	0.934
Left	Reference			
Treatment				
None	−1.85 (−2.247 to −1.452)	0.203	83.302	**<0.001**
First-line therapy	−1.445 (−1.799 to −1.09)	0.181	63.826	**<0.001**
Naive second-line therapy	−1.387 (−1.898 to −0.877)	0.260	28.373	**<0.001**
Switch to second-line therapy	Reference			
Age	0.01 (−0.008 to 0.028)	0.009	1.295	0.255
Baseline P100 latency (ms)	0.019 (0.008–0.03)	0.006	11.092	**0.001**
Baseline amplitude (N75-P100)	0.014 (−0.036 to 0.064)	0.025	0,313	0.576
Baseline amplitude (P100-N145)	−0.041 (−0.091 to 0.009)	0.026	2.594	0.107

VEP, visual evoked potentials; EDSS, Expanded Disability Status Scale, SD, Standard deviation. Generalized Estimating Equations, β1: Unstandardized beta coefficient. Bold values indicate statistical significance at *p* < 0.05.

**Figure 2:**
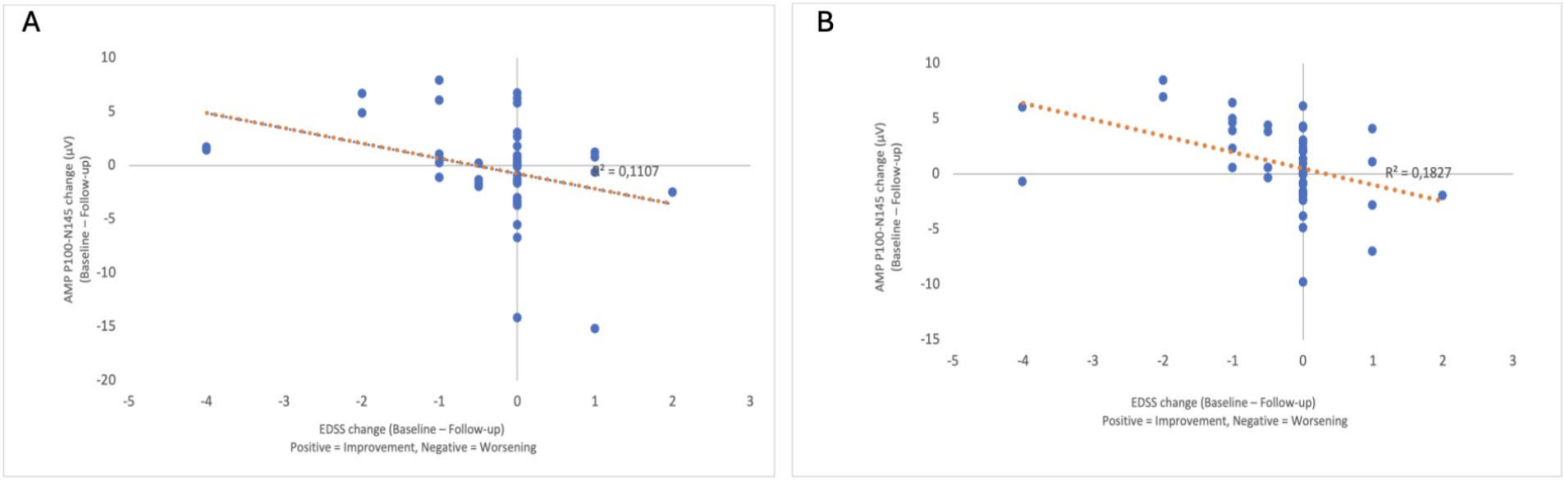
Scatter plots showing the relationship between the EDSS change (baseline—follow-up) (x-axis) and the P100–N145 amplitude change (baseline—follow-up) (y-axis) for the right eye **(A)** and the left eye **(B)**. The direction of the x-axis is indicated, with positive values representing EDSS improvement and negative values representing EDSS deterioration. A negative correlation was observed in both eyes, indicating that greater amplitude reduction was associated with greater EDSS progression.

Regarding the associations with EDSS worsening, the logistic GEE analysis showed that a one-unit increase in the P100–N145 amplitude difference was associated with a 1.511-fold rise in the odds of progression (OR: 1.511, 95% CI: 1,263–1,809, *p* < 0.001) (Table 3). Partial correlation analysis, adjusting for age, revealed a significant negative correlation between changes in EDSS and P100–N145 amplitude in both eyes, indicating that greater reductions in amplitude were associated with greater disability worsening (right eye: *r* = −0.339, *p* = 0.024; left eye: *r* = −0.406, *p* = 0.006) (Table 4). Scatter plots illustrate the relationship between the change in P100–N145 amplitude and the change in EDSS (baseline—follow-up) (Figures 2A,B).

**Table 3:** Binary logistic regression analysis results for EDSS worsening.

	Multiple	
	OR (%95 CI)	*p*
Constant	0.109 (0.006–1.998)	0.135
Eye		
Right	1.524 (0.402–5.782)	0.535
Left	Reference	
Age	0.989 (0.928–1.054)	0.730
P100 latency difference (ms)	0.983 (0.93–1.038)	0.533
Amplitude difference (N75–P100)	1.005 (0.816–1.238)	0.960
Amplitude difference (P100–N145)	1.511 (1.263–1.809)	**<0.001**

EDSS, Expanded Disability Status Scale. OR (95% CI), Odds ratio 95% confidence interval. Bold values indicate statistical significance at *p* < 0.05.

**Table 4:** Correlation between eye-specific VEP parameter changes and EDSS difference.

Eye	Parameter difference (baseline—follow-up)	EDSS difference	
		*r*	*p*
Right	P100 latency difference (ms)	−0.035	0.822^x^
	Amplitude difference (N75–P100)	−0.143	0.354^x^
	Amplitude difference (P100–N145)	−0.339	**0.024** ^x^
Left	P100 latency difference (ms)	−0.001	0.994^x^
	Amplitude difference (N75–P100)	−0.108	0.483^x^
	Amplitude difference (P100–N145)	−0.406	**0.006** ^x^

EDSS, Expanded Disability Status Scale. ^x^Partial correlation was performed with age as the control variable. Bold values indicate statistical significance at *p* < 0.05.

A separate GEE model tested whether baseline VEP and demographic characteristics predicted EDSS changes, but no significant associations were found (all *p* > 0.05) (Table 5).

**Table 5:** Baseline clinical and electrophysiological predictors of disability progression.

	β1 (%95 CI)	SD	Test statistic	*p*
Constant	1.903 (−0.737 to 4.543)	1.347	1.996	0.158
Eye				
Right	−0.027 (−0.398 to 0.344)	0.189	0.021	0.885
Left	Reference			
Treatment				
None	0.614 (−0.203 to 1.432)	0.417	2.169	0.141
First-line therapy	0.403 (−0.335 to 1.14)	0.376	1.144	0.285
Naive second-line therapy	0.289 (−0.584 to 1.161)	0.445	0.42	0.517
Switch to second-line therapy	Reference			
Age	−0.029 (−0.063 to 0.004)	0.017	2.999	0.083
Baseline P100 latency (ms)	−0.009 (−0.018 to 0.001)	0.005	2.829	0.093
Baseline amplitude (N75-P100)	−0.014 (−0.112 to 0.085)	0.050	0.074	0.786
Baseline amplitude (P100-N145)	−0.044 (−0.109 to 0.021)	0.033	1.735	0.188

Generalized estimating equations; β1, unstandardized beta coefficient.

### 3.3 Comparison between the treatment groups

When analyzing the changes in VEP parameters in the different treatment groups, the most significant reduction in P100–N145 amplitude was observed in patients switched to second-line therapy (−1.32 ± 5.31 μV). In comparison, there was an intermediate increase in patients receiving first-line therapy (+1.04 ± 4.18 μV). In second-line naive patients, the change was minimal (−0.16 ± 3.39 μV). These differences were not statistically significant (*p* = 0.204), but may reflect underlying differences in disease severity (Table 6).

**Table 6:** Comparison of differences in quantitative variables (baseline–follow-up) across treatment groups.

	None	First-line therapy	Naive second-line therapy	Switch to second-line therapy	Total	Test statistic	*p*
P100 latency difference (ms)	−4.75 ± 10.34	−4.18 ± 10.28	−0.95 ± 10.21	1.93 ± 17.07	−2.47 ± 11.6	2.910	0.406 ^x^
Amplitude difference (N75–P100)	0.49 ± 1.64	0.73 ± 4.09	−0.33 ± 2.58	−1.13 ± 3.38	0.17 ± 3.61	4.375	0.224 ^x^
Amplitude difference (P100–N145)	−0.45 ± 2.25	1.04 ± 4.18	−0.16 ± 3.39	−1.32 ± 5.31	0.32 ± 4.17	4.591	0.204 ^x^
EDSS changes	0 ± 0	−0.24 ± 0.76	−0.32 ± 1.38	−0.64 ± 1.54	−0.31 ± 1.07	7.249	0.064 ^x^

^xGeneralized estimating equations, mean ± standard deviation.^

## 4 Discussion

Despite the widespread use of VEPs to monitor demyelination in MS, the prognostic significance of later waveform components—particularly P100–N145 amplitude—has been relatively underexplored. Most studies have focused on early components such as P100 latency and N75–P100 amplitude, which reflect conduction delay and demyelination. For instance, Vecchio et al. demonstrated associations between P100 latency and N75–P100 amplitude and Multiple Sclerosis Severity Score (MSSS) in non-neuritic eyes, although only P100 latency was found to be an independent predictor in regression analyses (12). Similarly, Piedrabuena and Bittar associated prolonged P100 latency and reduced VEP amplitudes with higher EDSS scores and decreased RNFL thickness in relapsing–remitting MS, but did not evaluate the P100–N145 segment or report longitudinal changes (13). Other studies have incorporated VEPs into multimodal systems combining magnetic resonance imaging (MRI) and optical coherence tomography (OCT) and have shown their utility in detecting subclinical damage and correlating with functional outcomes such as visual acuity and cognitive performance (14–17).

Extending these findings, our results show that a longitudinal decrease in P100–N145 amplitude correlates more strongly with EDSS progression than previous VEP parameters, highlighting its potential as a complementary electrophysiological marker for the accumulation of long-term disability. Notably, the change in P100–N145 amplitude was not predictive of future disability when measured at disease onset, but rather reflected concurrent functional deterioration, suggesting that amplitude may be a marker rather than a predictor of disease progression. This novel contribution emphasizes the need to include P100–N145 amplitude as a missing piece of the puzzle in MS monitoring and to refine its role in disease assessment.

### 4.1 A shift from demyelination to neurodegeneration

Previous studies have predominantly reported a correlation between P100 latency and EDSS progression, highlighting latency as an important marker of disability (18, 19). Although our study did not confirm this correlation, this does not necessarily contradict previous findings but suggests that latency alone may not consistently capture the long-term dynamics of disability. However, we observed a statistically significant cross-sectional correlation between P100 latency at baseline and EDSS score. Although the effect size was minimal, limiting the independent clinical utility, this modest association may suggest a residual relationship between conduction delay and overall disability burden at a given time point.

The significant negative correlation between the reduction in P100-N145 amplitude and worsening EDSS score in our study is consistent with the understanding that the reduction in amplitude reflects axonal loss, which is a key determinant of neurodegeneration in MS (20). Our results reinforce the notion that as MS progresses, axonal loss increasingly contributes to disability and complements, rather than replaces, the role of demyelination. This highlights the importance of considering both latency and amplitude as part of a comprehensive approach to disease monitoring.

### 4.2 Why P100–N145 amplitude and not N75–P100?

An important finding of our study was the significant negative correlation between P100 and N145 amplitude reduction and EDSS score deterioration, while N75–P100 amplitude showed no such correlation. Of note, the N75–P100 amplitude was associated with the initial EDSS score at baseline, suggesting that it is important in capturing early disability. This raises the important question: Why is P100–N145 more sensitive to disease progression than N75–P100?

The decision to focus on the P100–N145 amplitude rather than the N75–P100 segment when assessing VEPs in MS is based on the different neurophysiological processes that these components represent. While the N75–P100 interval primarily reflects early afferent conduction through the visual pathways and is sensitive to demyelination, the P100–N145 segment appears to capture later cortical processing involving higher-order visual areas, as shown in basic source localization studies (21, 22). Using simultaneous electroencephalography and functional MRI with dynamic causal modeling, Youssofzadeh et al. have shown that signal propagation continues beyond V1 into the dorsal stream regions, suggesting that the later VEP components reflect integrative cortical activity rather than primary conduction delays (23). In addition, Klistorner et al. linked delayed or reduced later VEP components to axonal injury, supporting the hypothesis that P100–N145 amplitude is more indicative of axonal integrity and neurodegeneration (24).

In contrast to latency, which primarily measures signal transmission speed, the amplitude reduction reflects declining neuronal integrity, synaptic dysfunction, and axonal loss—all critical aspects of MS pathology (25). In our study, the P100–N145 interval was found to be the VEP parameter most strongly associated with EDSS progression, indicating its potential value as a marker of functional deterioration in MS. This observation is supported by recent preclinical research emphasizing the importance of the later components of the VEP waveform in reflecting cortical dysfunction and neurodegenerative processes even in early disease stages (26). Taken together, these findings suggest that beyond early latency shifts, broader temporal dynamics within the VEP signal may provide greater sensitivity in tracking disease progression. The reduction in N75–P100 amplitude may be more pronounced in the early stages of MS due to acute demyelination, but may not adequately capture long-term neurodegenerative processes. On the other hand, the decline in P100–N145 amplitude appears to be more closely associated with progressive neurodegeneration, which is a stronger determinant of disability accumulation over time. This difference explains why P100–N145 amplitude correlates more strongly with EDSS progression than N75–P100.

### 4.3 Clinical implications: refining the role of the VEP in MS monitoring

Traditionally, MS progression has been monitored using clinical scales such as the EDSS, which, being an ordinal scale with limited sensitivity, may fail to capture subtle early neurodegenerative changes (27). In our cohort, the degree of EDSS change was relatively modest, which may have influenced the strength of the observed correlations. Nevertheless, our results support the potential of VEP, particularly the P100–N145 amplitude, as an early marker of disease progression that could complement standard clinical assessments. Identifying patients at risk of disability worsening before it becomes clinically apparent could enable more proactive treatment strategies and timely interventions.

In addition to our primary results, we also investigated how changes in VEP parameters varied with DMT status to gain initial insights into treatment response and disease behavior. Although the observation period was insufficient to assess long-term treatment efficacy, we believe that the amplitude changes observed in the different treatment subgroups may reflect inter-individual differences in the natural history of the disease. In particular, patients switched to second-line therapies showed a greater reduction in P100–N145 amplitude, whereas patients receiving first-line therapies showed relative stability or even an increase. Although these differences did not reach statistical significance, they may reflect variability in individual disease progression and emphasize the potential role of VEP amplitude in detecting subclinical progression, consistent with previous observations of differential treatment effects in MS pathophysiology and disease activity patterns (28).

### 4.4 Future directions: toward a multimodal approach

Given these findings, future research should investigate the integration of VEP amplitude measurements with other biomarkers, such as OCT and MRI-based atrophy metrics. A multimodal approach combining electrophysiology, structural imaging, and functional assessments could provide a more comprehensive understanding of MS pathology and improve early detection of disease progression. In the present study, structural imaging data were not available for comparison, which represents a limitation.

Additionally, both eyes were included in the analysis to more accurately reflect the bilateral nature of visual pathway involvement in MS. Given that subclinical demyelinating lesions can affect one eye without overt symptoms, limiting the analysis to a single eye might result in an incomplete assessment of disease burden.

Further studies should also investigate whether P100–N145 amplitude is particularly valuable in specific MS subtypes or disease stages to enhance its applicability in personalized treatment planning.

## Data Availability

The raw data supporting the conclusions of this article will be made available by the authors, without undue reservation.
